# Induced Abortion, Birth Control Methods, and Breast Cancer Risk: A Case-Control Study in China

**DOI:** 10.2188/jea.JE20170318

**Published:** 2019-05-05

**Authors:** Xuelian Yuan, Fang Yi, Can Hou, Hui Lee, Xiaorong Zhong, Ping Tao, Hui Li, Zhuping Xu, Jiayuan Li

**Affiliations:** 1 Department of Epidemiology and Biostatistics, West China School of Public Health, Sichuan University, Chengdu, Sichuan, China; 2 Department of Epidemiology and Biostatistics, School of Public Health, Southwest Medical University, Luzhou, Sichuan, China; 3 Department of Head and Neck and Mammary Gland Oncology, and Medical Oncology, Cancer Center, West China Hospital, Sichuan University, Chengdu, Sichuan, China; 4 Department of Breast Surgery, the Second People’s Hospital of Sichuan Province, Chengdu, Sichuan, China; 5 Shuangliu Maternal and Child Health Hospital, Chengdu, Sichuan, China

**Keywords:** breast cancer, induced abortion, oral contraceptive, intrauterine devices, case-control study

## Abstract

**Background:**

The association between induced abortion and birth control methods (including oral contraceptives and intrauterine devices) and breast cancer may vary among countries, due to the different usage and frequency of birth control methods and induced abortion among countries. A better understanding of this association may help in determining safer birth control methods for Chinese women.

**Methods:**

A case-control study was conducted with a total of 794 cases and 805 controls. Standardized questionnaires were used to collect information on demographic characteristics, exposure to induced abortion, birth control methods, and other risk factors for breast cancer. Multivariate logistic regression was conducted to explore the association between birth control methods and breast cancer.

**Results:**

Multivariate logistic regression analyses showed that having a history of medical abortions, ≥3 surgical abortions, or both medical and surgical abortions was associated with an increased risk of breast cancer in post-menopausal women (odds ratio [OR] 2.48; 95% confidence interval [CI], 1.14–5.40). Pre-menopausal women who had used intra-uterine devices (IUDs) for more than 20 years tended to have a lower breast cancer risk than other age-matched pre-menopausal women (OR 0.41; 95% CI, 0.25–0.68). Both pre-menopausal and post-menopausal women who had <20 years exposure to IUDs and those who had used two or more birth control methods (with the exception of women who used IUDs for more than 20 years) tended to have much higher breast cancer risk.

**Conclusion:**

The relationship between induced abortion and birth control methods and breast cancer was complex, though being exposed to induced abortion and two or more birth control methods in one’s lifetime appeared to be risk factors for breast cancer in Chinese women.

## INTRODUCTION

Breast cancer is the most common malignancy in Chinese women, whose incidence rate had risen from 23.37 per 100,000 in 2007 to 28.42 per 100,000 in 2013.^1^ In addition, the mean age at the time of breast cancer diagnosis in Chinese women is decreasing. A multicenter clinical epidemiological study showed that the peak age of onset for Chinese female patients was 50–65 years, about 10 years earlier than that among women in western countries.^2^ Not coincidentally, women of this age were generally at reproductive age during the period when the Chinese birth-control policy (one child per family) was fully implemented.

Breast cancer is estrogen-related, and it is believed that the policy of one child per family could be partially responsible for the increased incidence of breast cancer among Chinese women.^3^ Women who already had one child need to use birth control methods for a long period of time, and in some cases, women had induced abortion when other birth control methods failed. According to the China Health Statistics Yearbook 2016, the most common birth control method for Chinese women at childbearing age is induced abortion (IA), including surgical abortion (SA) and medical abortion (MA). In 2015, an estimated 9,851,961 women underwent IA in China, corresponding to a rate of 32.64 per 1,000 women aged 15 to 44.^4^ Use of an intrauterine device (IUD) is the second most common method, with an estimated 8,227,879 women having IUDs inserted in 2015 (30.81 per 1,000 women aged 20 to 44).^5^ Tcu220C is the most frequently used IUD type in China.^6^ The oral contraceptive (OC) usage rate among women of reproductive age in China is very low. In 2010, this prevalence rate was estimated to be 0.98%,^7^ which is much lower than that among American women aged 15–44 years.^8^ Some studies have focused on the relationships of the above risk factors with breast cancer in southeast China, but in mid-western China, where the one child birth control policy was implemented most stringently, there have been few relevant studies. Furthermore, Chinese women may use more than one birth control method over the course of their reproductive years, which leads to complex combinations of these exposures, making the relationships between birth control methods and risk of breast cancer difficult to discern. Zhen et al^9^ and Shi et al^10^ both found that there are positive relationships of MA and OC with breast cancer, while studies conducted by Wu et al^11^ and Li et al^12^ indicated no such relationship. As for studies focusing on IUD and breast cancer risk, few have been conducted in Chinese women.

This study is a matched case-control study conducted in Chengdu, with the purpose of exploring the effect of common birth control methods and IA on breast cancer in Chinese women. As a central city in southwest China, Chengdu is representative of the area in terms of economic level as well as composition of the population. In this study, breast cancer cases were recruited from three government-owned hospitals in Chengdu: Sichuan Provincial People’s Hospital, Sichuan Cancer Hospital, and West China Hospital. These three hospitals have reputations for providing high-quality cancer treatment, so patients come to them from across the southwest. Hence, the cases we recruited are representative of breast cancer patients from southwest China.

## MATERIALS AND METHODS

### Study subjects

This was a matched case-control study with 794 cases and 805 controls. For the case group, 794 female patients with newly histopathologically diagnosed primary breast cancer were recruited from April 2014 through May 2015 from the hospitals listed above. Other inclusion criteria were Han nationality, had lived in Sichuan for more than 3 years, and had no history of mental disorder. We further excluded patients with metastatic breast cancer, as they had a low response rate. Among the 794 cases recruited, 424 (53.4%) were city dwellers and 370 (46.6%) lived in rural areas. For each case, a residence area-matched (urban or rural) female control was recruited from women who underwent annual physical examinations in two physical examination centers located in Wuhou district and Shuangliu county in Chengdu, to account for lifestyle differences caused by social economic level.^13^ Controls were recruited during the same period of time and were eligible if they had no previous diagnosis of malignancy or mental disorder, were confirmed breast cancer-free via breast ultrasound and mammography, had Han nationality, and had lived in Sichuan for more than 3 years. The numbers of urban and rural residents in our control group were 384 (47.7%) and 421 (52.3%), respectively.

The study protocol was approved by the ethics committee of Sichuan University. All subjects participated in the study voluntarily and signed informed consent forms.

### Variable definition

#### Menopausal status

Women who met any of the following criteria were classified as post-menopausal: 1) absence of menstruation for at least 1 year (any age); 2) bilateral oophorectomy or estrogen deprivation therapy; 3) if there is not information on menstruation history, older than 60 years.

#### Induced abortion (IA) exposure

Women who had at least one IA were categorized as having IA exposure.

#### Oral contraceptive (OC) exposure

Previous studies have found that OC may increase the risk of breast cancer, but there was no statistical difference in breast cancer risk between women who have not taken OC for the past 10 or more years and those who have never taken it, indicating that the effect of OC exposure on breast cancer risk may fades with withdrawal time.^14^^,^^15^ Therefore, we grouped women who have taken OC at any point within the past 10 years as OC users, and all others as non-users.

#### Intrauterine device (IUD) exposure

Women who had had an IUD implanted for at least 1 year were defined as having IUD exposure.

#### Body Mass Index (BMI)

BMI was defined as weight (kg)/height (m)^2^. We used 24.0 as the cutoff value for BMI, in accordance with the WGOC and The International Life Sciences Institute China Office.^16^

#### History of estrogen-related disease

Women who had previous diagnosis of diseases related to increased estrogen exposure, such as breast diseases, ovaries diseases, uterus diseases, and other chronic diseases (hypertension, diabetes, and chronic cholecystitis), were defined as having a history of estrogen-related disease.

#### Active smoking

Active smoking was defined as smoking for more than 6 months (including former smokers).

#### Passive smoking

We defined passive smoking as non-smokers who were exposed to tobacco smoking for more than 15 minutes at least 1 day per week.

#### Alcohol drinking

We defined alcohol drinking as participants who drunk alcohol at least one time each week.

#### Physical activity

We used the following formula from the Compendium of physical activities^17^ to calculate the amount of physical activity in the recent five years (excluding occupational physical activity):

$$\begin{array}{l} \text{Metabolic equivalent }(MET)-hour/week\\ \;=\sum MET_{i}\times hour_{i}\;per\;week \end{array}$$

MET is defined as the ratio of metabolic rate during physical activity to a standard resting metabolic rate.^17^ The median MET in the control group was used as the cutoff value for categorization of high physical activity (>median MET) and low physical activity (≤median MET).

### Data collection

A structured questionnaire was used to collect information on demographics and risk factors for breast cancer, including general demographic characteristics (age, education, career, health insurance, and family per capita income), BMI (calculated from height and weight), reproductive history (menopausal status, menarche age, menopause age, pregnancy times, number of live birth, age at first delivery, number and the reason of spontaneous abortion, and breastfeeding duration per child), history of estrogen-related diseases, family history of cancer (breast/ovaries/uterus/other cancers of relatives, relationship between patients and relatives, name of other cancer, and onset age) and lifestyles (active smoking, passive smoking, alcohol consumption, and physical activity: physical exercise types, start-stop year, frequency of exercise, and exercise hours per session). We also collected information on exposure to the following birth control methods in detail:

IA: the number of medical abortions and surgical abortions.
OC: the most recent OC use was collected to categorize women into OC users and non OC users. Women who had used OC in the last 10 years were defined as OC users, and others were defined as non-users. For OC users, further information on the types of OC used was collected, including long acting contraceptives (LOC), short acting contraceptives (SOC), and emergency contraceptives (EOC). As different types of OC have different components, their effects on breast cancer risk can be different. At present, the main components of LOC and SOC are estrogen and progesterone, while EOC contains progesterone only. Therefore, we classified OC exposure as either LOC or SOC exposure or as EOC exposure.
IUD: the approximate dates of placing and removing the IUD were collected to calculate the total exposure time.

All the information was collected by trained interviewers using in-person interviews. Data were entered into Epidata 3.0 (The Epidata Association, Odense, Denmark) and analyzed using SPSS 20.0 (IBM Corp, Armonk, NY, USA).

### Data analysis

Statistical analyses of pre-menopausal women were conducted separately from post-menopausal women. The Student *t* test or Chi-square test was used to describe and compare the distribution of demographic characteristics and breast cancer risk factors between cases and controls. Variables that were found to be statistically different between two groups were selected as covariates to be adjusted in the multivariate logistic regression. We further adjusted age in both the analyses of pre-menopausal and post-menopausal women. The key point of our analysis was clarifying the criteria of exposure to birth control methods. Based on the results of preanalysis and our previous research,^18^ we determined the cutoff values of times of IA use, times of OC use, and total time of IUD use. Then, in accordance with the results, women who met all the following conditions were defined as the reference group: never had IA or just had SA ≤2 times, and never used OC or IUD, and multivariate logistic regression was performed to explore the separate and combined effects of IA, OC, and IUD use on breast cancer risk and to calculate odds ratios (ORs) with 95% confidence intervals (CIs) after adjusting for confounders. Moreover, the dose-response relationship of IUD placing time was estimated using the Chi-square test for trend. Statistical tests were based on two-sided probability with a significance level of 0.05.

## RESULTS

### Distribution and comparison of demographic and reproductive factors between cases and controls in different menopausal states

Among the 1,599 subjects, 448 cases and 463 controls were pre-menopausal women with average ages of 43.71 (standard deviation [SD], 6.13) and 43.35 (SD, 5.43) years, respectively, and 346 cases and 342 controls were post-menopausal women with average ages of 58.55 (SD, 6.87) and 56.60 (SD, 6.44) years, respectively. Chi-square tests and Student *t* tests showed that among pre-menopausal women, cases and controls were similar with the exception of per capita annual income, active smoking, and passive smoking (*P* < 0.05). Among post-menopausal women, cases and controls were significantly different in terms of their age, per capita annual income, active smoking, alcohol drinking and history of live birth (*P* < 0.05; eTable 1).

### Correlation between induced abortion and breast cancer

Among pre- or post-menopausal women, compared with those who never had IA, women who experienced at least one MA had a higher risk of breast cancer (OR 3.76; 95% CI, 1.39–10.19 for pre-menopausal women and OR 7.20; 95% CI, 1.30–39.98 for post-menopausal women; Table 1). As for post-menopausal women, those who had SA three or more times had an increased risk of developing breast cancer compared with women who never had IA (OR 2.06; 95% CI, 1.23–3.45; Table 1). Moreover, we found significant effects of exposure of MA and SA breast cancer risk among both pre-menopausal and post-menopausal women (OR 6.80; 95% CI, 2.22–20.85 for pre-menopausal women and OR 17.14; 95% CI, 1.96–149.89 for post-menopausal women; Table 1).

**Table 1.** Correlation between induced abortion and breast cancer risk

	Premenopausal women				Postmenopausal women			
	
	Case *N* (%)	Control *N* (%)	cOR^d^ (95% CI)	aOR^e^ (95% CI)	Case *N* (%)	Control *N* (%)	cOR^d^ (95% CI)	aOR^f^ (95% CI)
**Induced abortion^a^**								
Never	93 (20.8)	124 (26.8)	1	1	95 (27.5)	95 (27.8)	1	1
Ever	355 (79.2)	339 (73.2)	**1.40 (1.03–1.90)**	**1.34 (0.98–1.83)**	251 (72.5)	247 (72.2)	1.02 (0.73–1.42)	1.26 (0.88–1.79)
**Types of induced abortion used**								
None^b^	93 (20.8)	124 (26.8)	1	1	95 (27.5)	95 (27.8)	1	1
MA	15 (3.3)	6 (1.3)	**3.33 (1.25–8.92)**	**3.76 (1.39–10.19)**	8 (2.3)	2 (0.6)	4.00 (0.83–19.33)	**7.20 (1.30–39.98)**
SA	321 (71.7)	329 (71.1)	1.30 (0.95–1.77)	1.23 (0.89–1.68)	234 (67.6)	244 (71.3)	0.96 (0.69–1.34)	1.09 (0.75–1.58)
MA+SA^*^	19 (4.2)	4 (0.9)	**6.33 (2.09–19.24)**	**6.80 (2.22–20.85)**	9 (2.6)	1 (0.3)	**9.00 (1.12–72.44)**	**17.14 (1.96–149.89)**
**Number of surgical abortion^c^**								
0	93 (22.5)	124 (27.4)	1	1	95 (28.9)	95 (28.0)	1	1
1	123 (29.7)	135 (29.8)	1.22 (0.85–1.75)	1.13 (0.78–1.64)	88 (26.7)	112 (33.0)	0.79 (0.53–1.17)	0.81 (0.51–1.29)
2	106 (25.6)	110 (24.3)	1.29 (0.88–1.89)	1.25 (0.85–1.83)	72 (21.9)	83 (24.5)	0.87 (0.57–1.33)	0.97 (0.62–1.1.50)
≥3	92 (22.2)	84 (18.5)	1.43 (0.93–2.21)	1.33 (0.88–2.00)	74 (22.5)	49 (14.5)	1.51 (0.95–2.39)	**2.06 (1.23–3.45)**

^a^A total of 1,599 subjects.

^b^A total of 438 subjects who never had induced abortion or only had medical abortion.

^c^A total of 1,535 subjects who never had IA or only had SA.

^d^cOR: crude OR, it is an OR value estimated by unconditional logistic regression with non-confounders adjusted.

^e^aOR: adjusted OR, it is an OR value estimated by multivariate logistic regression with age, per capita annual income, history of active smoking, passive smoking, and pregnant times.

^f^aOR: adjusted OR, it is an OR value estimated by multivariate logistic regression with age, per capita annual income, history of active smoking, alcohol drinking, history of live birth, and pregnant times adjusted.

^*^Women who have exposure to both of Medical abortion and Surgical abortion in their lifetime.

### Correlation between OC and breast cancer

Among pre-menopausal women, OC users had significantly higher odds of being diagnosed with breast cancer than non-users (OR 2.06; 95% CI, 1.39–3.04; Table 2). This association was found in both LOC/SOC users (OR 2.00; 95% CI, 1.19–3.36) and EOC users (OR 2.13; 95% CI, 1.24–3.66). The effect of OC was only found in short term pre-menopausal users (1 to 6 months) (OR 2.69; 95% CI, 1.22–5.94; Table 2), and EOC users who had EOC more than 2 times (OR 2.45; 95% CI, 1.11–5.41).

**Table 2.** Correlation between OC use and breast cancer risk

	Premenopausal women				Postmenopausal women			
	
	Case *N* (%)	Control *N* (%)	cOR^d^ (95% CI)	aOR^e^ (95% CI)	Case *N* (%)	Control *N* (%)	cOR^d^ (95% CI)	aOR^f^ (95% CI)
OC use^*a^								
Never	362 (80.8)	414 (89.4)	1	1	318 (91.9)	323 (94.4)	1	1
Ever	86 (19.2)	49 (10.6)	**2.01 (1.38–2.93)**	**2.06 (1.39–3.04)**	28 (8.1)	19 (5.6)	1.50 (0.82–2.74)	1.41 (0.76–2.64)
**Types of OC use^a^**								
None	362 (80.8)	414 (89.4)	1	1	318 (91.9)	323 (94.4)	1	1
LOC/SOC	46 (10.3)	25 (5.4)	**2.10 (1.27–3.49)**	**2.00 (1.19–3.36)**	21 (6.1)	13 (3.5)	1.78 (0.86–3.67)	1.59 (0.70–3.58)
EOC	40 (8.9)	24 (5.2)	**1.91 (1.13–3.22)**	**2.13 (1.24–3.66)**	7 (2.0)	6 (1.8)	1.19 (0.39–3.57)	1.19 (0.34–4.10)
**Time of LOC or SOC use, months^b^**								
None	402 (89.7)	438 (94.6)	1	1	325 (93.9)	329 (96.2)	1	1
1–6	24 (5.4)	9 (1.9)	**2.91 (1.34–6.33)**	**2.69 (1.22–5.94)**	10 (2.9)	4 (1.2)	2.53 (0.79–8.16)	2.81 (0.73–10.76)
7–12	11 (2.5)	7 (1.5)	1.71 (0.66–4.46)	1.81 (0.69–4.75)	4 (1.2)	1 (0.3)	4.05 (0.45–36.42)	2.75 (0.22–34.59)
13–39	4 (0.9)	5 (1.1)	0.87 (0.23–3.27)	0.68 (0.17–2.76)	3 (0.9)	3 (0.9)	1.01 (0.20–5.05)	1.50 (0.27–8.17)
39	7 (1.6)	4 (0.9)	1.91 (0.55–6.56)	1.66 (0.47–5.84)	4 (1.2)	5 (1.5)	0.81 (0.22–3.04)	0.56 (0.11–2.80)
**Number of times per year EOC was used^c^**								
None	402 (89.7)	434 (93.7)	1	1	339 (98.0)	335 (98.0)	1	1
≤2	27 (6.0)	19 (4.1)	1.53 (0.84–2.80)	1.62 (0.87–3.01)	6 (1.7)	5 (1.5)	1.19 (0.36–3.92)	1.19 (0.35–4.04)
2	19 (4.2)	10 (2.2)	2.05 (0.94–4.46)	**2.45 (1.11–5.41)**	1 (0.3)	2 (0.6)	0.49 (0.05–5.48)	0.69 (0.06–7.87)

CI, confidence interval; EOC, an oral hormone drug used to practise contraception, and which was taken within 72 hours after unprotected sex or contraception failure; LOC, an oral hormone drug that inhibit ovulation and anti-implantation, and which was taken once a month; SOC, an oral hormone drug that inhibit ovulation and anti-implantation, and which was taken once a day.

^*^Subjects who have ever used OC in the past 10 years were defined as OC users.

^a^A total of 1,599 subjects.

^b^Include subjects who only used LOC or SOC, and the total time of LOC or SOC use was categorized into quartile based on control distribution.

^c^Include subjects who only used EOC as well as reported the frequency, and the number of times per year EOC was used was categorized based on median of control distribution.

^d^cOR: crude OR, it is an OR value estimated by unconditional logistic regression with non-confounders adjusted.

^e^aOR: adjusted OR, it is an OR value estimated by multivariate logistic regression with age, per capita annual income, and history of active smoking, passive smoking adjusted.

^f^aOR: adjusted OR, it is an OR value estimated by multivariate logistic regression with age, per capita annual income, history of active smoking, alcohol drinking, and history of live birth adjusted.

### Correlation between IUD and breast cancer

For pre-menopausal women, there was some indication that IUD use was associated with an increased risk of breast cancer, but the results were statistically insignificant after adjusting for confounders (Table 3). We further categorized subjects into six groups according to the total exposure time to IUD and found that there was no significant dose-response relationship between IUD use and breast cancer risk (*P*_trend_ > 0.05, Table 3). Using an IUD for 10 or fewer years might be a risk factor for breast cancer in pre-menopausal and post-menopausal women, although the results were marginally insignificant in post-menopausal women after adjusting for confounders. We also found that using an IUD for 15–20 years could be a risk factor for breast cancer (OR 1.58; 95% CI, 1.02–2.43 in pre-menopausal women and OR 1.97; 95% CI, 1.14–3.38 in post-menopausal women; Table 3). Interestingly, the results indicated that >20 years’ exposure to IUD decreased the risk of breast cancer for pre-menopausal women (OR 0.43; 95% CI, 0.27–0.67, Table 3).

**Table 3.** Correlation between IUD use and breast cancer risk

	Premenopausal women				Postmenopausal women			
	
	Case *N* (%)	Control *N* (%)	cOR^d^ (95% CI)	aOR^e^ (95% CI)	Case *N* (%)	Control *N* (%)	cOR^d^ (95% CI)	aOR^f^ (95% CI)
**IUD use^a^**								
Never	165 (36.8)	199 (43.0)	1	1	131 (37.9)	131 (38.3)	1	1
Ever	283 (63.2)	264 (57.0)	**1.29 (0.99–1.69)**	1.17 (0.88–1.55)	215 (62.1)	211 (61.7)	1.02 (0.75–1.39)	1.23 (0.88–1.71)
**Total time of IUD use, years^b^**								
0	165 (36.8)	199 (43.0)	1	1	131 (37.9)	131 (38.3)	1	1
0.1–5.0	43 (9.6)	18 (3.9)	**2.88 (1.60–5.19)**	**3.05 (1.64–5.67)**	33 (9.5)	18 (5.3)	**1.83 (0.98–3.42)**	1.81 (0.92–3.54)
5.1–10.0	53 (11.8)	34 (7.3)	**1.88 (1.17–3.03)**	**2.04 (1.23–3.37)**	20 (5.8)	13 (3.8)	1.54 (0.74–3.22)	2.03 (0.89–4.62)
10.1–15.0	49 (10.9)	42 (9.1)	1.41 (0.89–2.23)	1.43 (0.87–2.34)	31 (9.0)	36 (10.5)	0.86 (0.50–1.47)	0.96 (0.53–1.74)
15.1–20.0	79 (17.6)	61 (13.2)	**1.56 (1.06–2.31)**	**1.58 (1.02–2.43)**	55 (15.9)	31 (9.1)	**1.77 (1.07–2.93)**	**1.97 (1.14–3.38)**
>20.0	59 (13.2)	109 (23.5)	**0.65 (0.45–0.95)**	**0.43 (0.27–0.67)**	76 (22.0)	113 (33.0)	**0.67 (0.46–0.98)**	0.72 (0.47–3.38)
***P_trend_***^c^			1.07 (0.302)				2.27 (0.132)	

^a^A total of 1,599 subjects.

^b^A total of 1,599 subjects who had never used IUD or had complete information on total time of IUD use.

^c^It is a *P* value estimated by test of linear by linear Chi square test.

^d^cOR: crude OR, it is an OR value estimated by unconditional logistic regression with non-confounders adjusted.

^e^aOR: adjusted OR, it is an OR value estimated by multivariate logistic regression with age, per capita annual income, history of active smoking, passive smoking and amount of physical activity adjusted.

^f^aOR: adjusted OR, it is an OR value estimated by multivariate logistic regression with age, per capita annual income, history of active smoking, alcohol drinking and history of live birth adjusted.

### Effects of exposure of birth control methods and induced abortion on breast cancer occurrence

With possible confounders adjusted, multiple logistical regression showed that the odds of having breast cancer in post-menopausal women who either had MA only, had SA ≥3 times only, or were exposed to both MA and SA but no other methods of birth control, was 148% higher (OR 2.48; 95% CI, 1.14–5.40; Table 4), and a lower risk (OR 0.41; 95% CI, 0.25–0.68; Table 4) was found in pre-menopausal women who had >20 years of exposure to IUD compared with the reference group. Furthermore, in both pre-menopausal and post-menopausal women, those who had ≤20 years of exposure to IUD only and those exposed to two or more birth control methods (excluding subjects who used IUD for more than 20 years) were more likely to have breast cancer when compared with the reference group (Table 4).

**Table 4.** Correlation between induced abortion, OC, and IUD use and breast cancer risk

	Premenopausal women				Postmenopausal women			
	
	Case *N* (%)	Control *N* (%)	cOR^e^ (95% CI)	aOR^f^ (95% CI)	Case *N* (%)	Control *N* (%)	cOR^e^ (95% CI)	aOR^g^ (95% CI)
Reference group^a^	97 (22.3)	131 (28.7)	1.00	1.00	88 (26.7)	105 (31.9)	1.00	1.00
MA,^b^ MA+SA, or SA^c^ ≥3 times	36 (8.3)	32 (7.0)	1.52 (0.88–2.62)	1.50 (0.84–2.66)	25 (7.6)	14 (4.3)	**2.13 (1.04–4.35)**	**2.48 (1.14–5.40)**
OC use only	24 (5.5)	26 (5.7)	1.25 (0.68–2.30)	1.09 (0.54–2.20)	11 (3.3)	10 (3.0)	1.31 (0.53–3.24)	1.58 (0.58–4.33)
≤20 years IUD use only	133 (29.9)	105 (23.1)	**1.71 (1.19–2.47)**	**1.71 (1.15–2.56)**	93 (28.2)	73 (22.2)	**1.52 (1.00–2.31)**	**1.72 (1.08–2.73)**
>20 years IUD use only	46 (10.3)	99 (21.6)	**0.63 (0.41–0.97)**	**0.41 (0.25–0.68)**	58 (17.6)	98 (29.8)	0.71 (0.46–1.09)	0.85 (0.52–1.39)
Two or more methods used^d^	99 (22.8)	61 (13.4)	**2.19 (1.45–3.31)**	**2.35 (1.51–3.66)**	55 (16.7)	29 (8.8)	**2.26 (1.23–3.85)**	**3.49 (1.88–6.50)**

CI, confidence interval; IUD, contraceptive devices in the uterus used to practice contraception; OC, oral hormone drugs used to practice contraception.

^a^Including the subjects who had not used OC in the past 10 years, never used IUD, and either never had induced abortion or had surgical abortion ≤2.

^b^Medical abortion.

^c^Surgical abortion.

^d^Subjects who had used IUD for more than 20 years were excluded.

^e^cOR: crude OR, it is an OR value estimated by unconditional logistic regression with non-confounders adjusted.

^f^aOR: adjusted OR, it is an OR value estimated by multivariate logistic regression with age, per capital annual income, history of active smoking, and passive smoking adjusted.

^g^aOR: adjusted OR, it is an OR value estimated by multivariate logistic regression with age, per capita annual income, history of active smoking, alcohol drinking, and history of live birth adjusted.

## DISCUSSION

Our results indicate that having a history of MA, more than 3 times of SA, or both MA and SA was associated with an increased risk of breast cancer in post-menopausal women. Pre-menopausal women who had used IUD for more than 20 years tended to have a lower breast cancer risk than the rest pre-menopausal women. Both pre-menopausal and post-menopausal women who had <20 years of exposure to IUD and those who had used two or more birth control methods (with the exception of women who used IUD for more than 20 years) tended to have much higher breast cancer risk.

As a method of terminating pregnancy, IA includes both MA and SA. Only a few studies have specifically investigated the relationship between different types of IA and breast cancer risk, with conflicting results. One meta-analysis of fifteen prospective studies showed an insignificant association between breast cancer and IA (RR 1.00; 95% CI, 0.94–1.05),^19^ but the majority of the participants were western women, whose lifestyles and usage of birth control methods and IAs were different from Chinese women. Therefore, the results may have weak representativeness with respect to Chinese women. By contrast, a recent meta-analysis of 36 studies covering 14 Chinese provinces found that Chinese women with a history of IA had 49% higher odds of getting breast cancer than those with no history of IA (OR 1.49; 95% CI, 1.23–1.74), and the risk of breast cancer increased with the number of abortions.^20^ It is well known that women’s estrogen and progesterone levels increase during a pregnancy, which promotes the growth of the mammary glands. But when a pregnancy is terminated by SA, estrogen and progesterone levels suddenly drop, which consequently terminates the growth of breast cells, leading to acinar atrophy. Incompletely differentiated breast epithelial cells are more susceptible to stimulation by carcinogenic substances, and therefore become cancerous relatively easily.^21^^,^^22^ As for the relationship between MA and breast cancer, previous research showed that drug containing antiprogestin was used for terminating pregnancy at the early stages,^23^ and the antiprogestin contained in the drug may interfere with the internal hormone environment, making it conducive to the development of hormone-related tumors. Meanwhile, SA and MA can increase the risk of breast cancer by delaying the timing of a full-term pregnancy, which is considered to be a protective factor of breast cancer.^24^ In a departure from previous studies, we investigated the effects of MA and SA separately and found that having SA <3 times had no impact on breast cancer occurrence. However, for women with a history of both MA and SA, the risk of having breast cancer was 88% higher than for those exposed to MA or SA alone. But regretfully, the time of induced abortion was not included in the present study, which is meaningful and should be analyzed in later study. Our results indicated that it would be better for government to discourage IA as a method to control the birth number.

The relationship between OC and breast cancer is still inconclusive, although most studies have found that OC increases the risk of breast cancer. A case-control study of 200 cases and 403 controls indicated that OC non-users had a lower risk of breast cancer (OR 0.454; 95% CI, 0.234–0.879).^25^ A systematic review of 15 case-control studies and 8 cohort studies found that a history of OC use slightly increases breast cancer incidence (OR 1.08; 95% CI, 1.00–1.17), but the results were no longer significant when only United States-based studies were included (OR 1.03; 95% CI, 0.93–1.14).^26^ Two recent case-control studies conducted in Norway^27^ and Saudi Arabia^28^ both indicated that breast cancer risk increased only if OC use continued for at least 10 years.

Although the main components of OC are hormones, different types of OC have different components, so their effect on breast cancer may be different. However, none of the above studies distinguished LOC, SOC, and EOC, whereas in our study, OC users were further categorized as LOC/SOC users and EOC users. Our results showed that compared with women who did not have a history of OC use, pre-menopausal women with a history of LOC/SOC or EOC use were more susceptible to breast cancer. However, we did not find a statistically significant association between either LOC/SOC or EOC use history and breast cancer risk in post-menopausal women. This may be due to the low percentage of OC users in the controls; only 8% of the participants in the control group had a history of OC use. What’s more, the definitions of OC users and non-users were dependent only upon the last time subjects had taken OC, and had nothing to do with the dosage. Therefore, whether OC use increases the risk of breast cancer is not clear from this study.

The most common types of IUDs are T-shaped copper, ring, γ, and uterine cavity-shaped.^29^ Currently, T-shaped copper IUDs are the most commonly-used type among Chinese women.^6^ A survey about estrogen and breast cancer conducted in Finland found a positive association between breast cancer risk and previous hormonal IUD use in post-menopausal women (OR 1.48; 95% CI, 1.10–1.99),^30^ while a cohort study of 66,661 Chinese women in Shanghai found that more than 14 years’ exposure to IUDs may be a protective factor for breast cancer (HR 0.80; 95% CI, 0.62–1.02).^31^ We found that at least 20 years’ exposure to IUD was associated with decreased risk of breast cancer. A possible reason for this difference is that the previous studies did not rule out the effects of other birth control methods. The underlying mechanism of the protective effect of using an IUD for more than 20 years is still unclear, though Curtis indicated that IUD did not increase the risk of neoplasia.^32^ However, we also found placing IUD for less than 20 years was associated with an increased incidence of breast cancer. A possible explanation is that copper-containing IUDs were found to be related to a reduction in estrogen receptors in animal study,^33^ which could provide free estrogen for tumor formation. Furthermore, we also found that exposure to two or more birth control methods could be a risk factor for breast cancer. It is clear that IA, OC, and copper- or contraceptive-containing IUDs can affect breast cancer incidence by influencing the body’s estrogen level. There may be interactions between these methods in affecting breast cancer incidence, but the mechanism of these interactions remains unclear.

Compared with previous studies, exposure to different birth control methods was measured more precisely in our study. Ours was also the first study we know of to investigate the combined exposure of different birth control methods on breast cancer risk using women with relatively low risk as the reference group. Our study also has some limitations. Although we have matched the cases and controls by residence area, selection bias is still unavoidable due to the case-control study design. Second, we only collected information on the time of OC use, not dosage, so we could not explore the relationship between OC dosage and breast cancer risk. Moreover, due to the limited sample size of our study, we were not able to investigate any high-order interactions among birth control methods in breast cancer risk. Finally, our limited sample size may be partially responsible for our statistically insignificant results for some potential associations. Thus, cohort studies with detailed surveys and large sample sizes are needed to further investigate the relationship between birth control methods and breast cancer risk in Chinese women.

### Conclusion

Our results provide some epidemiological evidence that MAs, SAs, and less than 20 years IUD use could increase the risk of breast cancer for post-menopausal women. As for pre-menopausal women, less than 20 years IUD use could also increase the risk of breast cancer, while more than 20 years’ IUD use was associated with a decreased breast cancer risk. For both pre-menopausal and post-menopausal women who had been exposed to two or three kinds of birth control methods, the risk of having breast cancer was higher.

## APPENDIX A. SUPPLEMENTARY DATA

eTable 1. Comparison of demographic characteristics and risk factors between cases and controls
