# Supplementary material for: Induced Abortion, Birth Control Methods, and Breast Cancer Risk: A Case-Control Study in China
Source: J Epidemiol. 2019 May 5;29(5):173–9. doi: 10.2188/jea.JE20170318 (PMC6445797; doi:10.2188/jea.JE20170318)
Supplement: Supplementary file 1 [file je-29-173-s001.pdf]

eTable 1. Comparison of demographic characteristics and risk factors between cases and controls

	Premenopausal women			Postmenopausal women		
	Case N (%)	Control N (%)	*t(t')/χ^2 (corrected χ^2)(p-value)*	Case N (%)	Control N (%)	*t(t')/χ^2 (corrected χ^2)(p-value)*
Age, years						
Mean (SD)	43.71(6.13)	43.35(5.43)	-0.93 (0.361)	58.55(6.87)	56.60(6.44)	**-3.83 (<0.0001)**
Income, yuan						
<50,000	305 (68.1)	285 (61.6)	**4.25 (0.039)**	255 (73.7)	219 (64.0)	**7.50 (0.006)**
≥50,000	143 (31.9)	178 (38.4)		91 (26.3)	123 (36.0)	
BMI,[a] kg/m^2						
<24	299 (66.7)	286 (61.8)	2.45 (0.118)	206 (59.4)	191 (55.8)	0.90 (0.327)
≥24	149 (33.3)	177 (38.2)		140 (40.6)	151 (44.2)	
Active smoking						
No	432 (96.4)	458 (98.9)	**6.28 (0.012)**	332 (96.0)	340 (99.4)	**9.07 (0.003)**
Yes[b]	16 (3.6)	5 (1.1)		14 (4.0)	2 (0.6)	
Passive smoking						
No	197 (44.0)	254 (54.9)	**10.80 (0.001)**	178 (51.4)	198 (57.90)	2.89 (0.089)
Yes[c]	251 (56.0)	209 (45.1)		168 (48.6)	144 (42.10)	
Alcohol drinking[d]						
No	432 (96.4)	446 (96.3)	0.01 (0.935)	329 (95.1)	335 (98.0)	**4.20 (0.040)**
Yes	16 (3.6)	17 (3.7)		17 (4.9)	7 (2.0)	
Amount of physical activities[e]						
High	105 (23.44)	127 (27.43)	1.91 (0.167)	51 (14.74)	65 (19.01)	2.23 (0.135)
low	343 (76.56)	336 (72.57)		295 (85.26)	277 (80.99)	

Age at menarche, years						
≥14	214 (47.8)	238 (51.4)	1.20 (0.271)	252 (72.8)	230 (67.30)	2.55 (0.110)
<14	234 (52.2)	225 (48.6)		94 (27.20)	112 (32.70)	
History of live birth						
No	13 (2.9)	18 (3.9)		13 (3.80)	3 (0.90)	
Yes	435 (97.1)	445 (96.1)	0.67 (0.412)	333 (96.2)	339 (99.1)	**6.28 (0.012)**
History of estrogen-related disease						
No	259 (57.8)	288 (62.2)	1.83 (0.169)	217 (62.9)	232 (67.8)	1.99 (0.161)
Yes	189 (42.2)	175 (37.8)		129 (37.1)	110 (32.2)	
Family history of breast cancer						
No	433 (96.7)	454 (98.1)	1.75 (0.191)	332 (96.0)	333 (97.4)	1.07 (0.319)
Yes	15 (3.3)	9 (1.9)		14 (4.0)	9 (2.6)	

BMI, body mass index; SD, standard deviation.

[a] BMI was categorized according to the suggestion of WGOC

[b] Smoking for more than 6 months (include past smokers who smoked for more than 6 months)

[c] Non-smokers who were exposed to tobacco smoking for more than 15 minutes at least one day per week

[d] Drinking at least one times for a week

[e] The median MET in the control group was used as the cutoff value for categorization of high physical activity (>median MET) and low physical activity (≦median MET).
